# Glycosylation spectral signatures for glioma grade discrimination using Raman spectroscopy

**DOI:** 10.1186/s12885-023-10588-w

**Published:** 2023-02-21

**Authors:** Agathe Quesnel, Nathan Coles, Claudio Angione, Priyanka Dey, Tuomo M. Polvikoski, Tiago F. Outeiro, Meez Islam, Ahmad A. Khundakar, Panagiota S. Filippou

**Affiliations:** 1 School of Health & Life Sciences, Teesside University, TS1 3BX Middlesbrough, UK; 2 National Horizons Centre, Teesside University, 38 John Dixon Ln, DL1 1HG Darlington, UK; 3 School of Computing, Engineering & Digital Technologies, Teesside University, Darlington, UK; 4 Centre for Digital Innovation, Teesside University, Darlington, UK; 5 School of Pharmacy and Biomedical Sciences, University of Portsmouth, PO1 2UP Portsmouth, UK; 6 Translational and Clinical Research Institute, Faculty of Medical Sciences, Newcastle University, Newcastle upon Tyne, UK; 7 Department of Experimental Neurodegeneration, Center for Biostructural Imaging of Neurodegeneration, University Medical Center, Göttingen, Germany; 8 Max Planck Institute for Multidisciplinary Sciences, Göttingen, Germany; 9 Deutsches Zentrum für Neurodegenerative Erkrankungen (DZNE), Göttingen, Germany

**Keywords:** Raman spectroscopy, Gliomas, Biomolecular signatures, Diagnosis, Glioblastoma, Glycosylation

## Abstract

**Background:**

Gliomas are the most common brain tumours with the high-grade glioblastoma representing the most aggressive and lethal form. Currently, there is a lack of specific glioma biomarkers that would aid tumour subtyping and minimally invasive early diagnosis. Aberrant glycosylation is an important post-translational modification in cancer and is implicated in glioma progression. Raman spectroscopy (RS), a vibrational spectroscopic label-free technique, has already shown promise in cancer diagnostics.

**Methods:**

RS was combined with machine learning to discriminate glioma grades. Raman spectral signatures of glycosylation patterns were used in serum samples and fixed tissue biopsy samples, as well as in single cells and spheroids.

**Results:**

Glioma grades in fixed tissue patient samples and serum were discriminated with high accuracy. Discrimination between higher malignant glioma grades (III and IV) was achieved with high accuracy in tissue, serum, and cellular models using single cells and spheroids. Biomolecular changes were assigned to alterations in glycosylation corroborated by analysing glycan standards and other changes such as carotenoid antioxidant content.

**Conclusion:**

RS combined with machine learning could pave the way for more objective and less invasive grading of glioma patients, serving as a useful tool to facilitate glioma diagnosis and delineate biomolecular glioma progression changes.

**Supplementary Information:**

The online version contains supplementary material available at 10.1186/s12885-023-10588-w.

## Background

Gliomas encompass tumours of glial cell origin arising in the central nervous system. The highest grade of adult astrocytic glioma, glioblastoma (GBM), represents the most common and lethal form of brain tumour. GBM is characterized by hypoxia-driven necrosis, microvascular proliferation, and diffuse infiltration of cancer cells that migrate beyond the radiologically-defined tumour margins [1], making its treatment difficult [2]. Gliomas can be classified traditionally into four histological grades: non-malignant grades I (pilocytic) and II (diffuse), and malignant grades III (anaplastic) and IV (GBM) [3]. Since recently (2021 WHO Classification), the isocitrate dehydrogenase (IDH) genotype plays a more crucial role in the classification of glioma than histology alone [4]. Grade I histological glioma rarely progresses into higher-grades and is easily histologically distinguished [5]. However, higher-grade gliomas, and especially the two malignant grades (III and IV), are more challenging to discriminate as they may share overlapping characteristics [6].

Current glioma diagnosis relies on subjective histological assessment by a pathologist using immunohistochemical approaches, in addition to molecular characterization. Thus, there is a need for more objective and label-free diagnostic techniques, which may offer greater convenience, efficiency and value for money in the diagnosis of glioma [7]. Confocal Raman spectroscopy (RS), which combines vibrational spectroscopy with confocal microscopy, permits fingerprinting a sample’s chemical structure by analysing its biocomponents’ molecular bond vibrations. This information is represented as a spectral signature [8]; typically, a reduced spectral intensity correlates with a reduced concentration of biomolecules. RS has been used in cancer diagnosis to capture subtle changes in biomolecular composition, such as in DNA or protein [9], allowing comparison between cancerous and non-cancerous tissues and between stages of cancer development. RS has already been used in several areas of diagnosis [10], by accurately predicting the type and grade of cancerous tissue [11, 12]. Glioma serum samples have been successfully discriminated with RS from normal samples and other types of cancer [13–15]. In addition, normal brain, meningioma, glioma, and brain cancer metastasis tissues have been discriminated in formalin-fixed paraffin-embedded (FFPE) tissues [16] and low-grades from higher malignant glioma grades in serum samples [17].

More recently, RS has also been used to monitor protein post-translational modifications (PTMs) [18]. Modifications in the glycosylation patterns are highly transformed in cancer and are thought to play a key role in cancer development and progression [19]. Of note, some O-glycosylated proteins, including mucins found in serum, are overexpressed in cancer [20], suggesting a potential role as biomarkers [21]. Thus, there is considerable clinical potential in the detection of aberrant glycosylation patterns in the brain tissue and body fluids for the diagnosis, follow-up, and possibly even treatment of brain cancer [22].

In the present study, we used RS, in combination with machine learning approaches, to detect changes at different glioma grades, focussing on glycosylation patterns among the investigation of other biomolecular signatures. This study discriminated glioma grades with accuracy, and delineated biomolecular changes during glioma progression at three levels: (i) the tissue, comprising the tumour and the complex tumour microenvironment, (ii) moving to the circulation at the serum level, that can be exploited for liquid biopsy investigation and, (iii) at the single cell and multicellular spheroid level for further investigation of the glioma progression.

## Methods

### Patients and clinical samples

The Research Ethics Boards approved sample collection of the respective Biobanks (Manchester Cancer Research Centre (MCRC) (REC Ref 18/NW/0092) and NovoPath Biobank Newcastle (REC Ref 17/NE/0070)) UK and the Teesside University Research Ethics Committee upon receipt of the ethical approval. Informed consent was collected for each patient and all procedures followed the Declaration of Helsinki. Patient-informed consent was provided under the existing ethics approval procedures.

Serum samples were stored at − 80 °C prior to analysis. FFPE samples, cut on stainless-steel slides for Raman acquisitions and on glass for parallel histological analysis, were obtained from tumour debulking surgery or biopsies. Thirty FFPE tissue slides (10 grade II, 10 grade III, 10 grade IV) were obtained from NovoPath Biobank (Newcastle, UK). All grade II and grade III patients had a glioma of astrocytic type. Grades for each case were determined by a neuropathologist beforehand, following the histological classification. Thirty blood serum samples (10 non-glioma benign (controls), 10 grade III, 10 grade IV) were obtained from the MCRC biobank (Manchester, UK). Grade III patients of the serum sample set contained both oligodendrogliomas and astrocytes. Patients’ characteristics are summarized in Table S1, Supplementary file.

### In-house glycosylation database

To generate the glycosylation database, Raman spectra of glycan standards were collected. Glycan standards were mannose, fucose, N-acetyl-galactosamine, N-acetyl neuraminic acid, galactose, glucose, and N-acetyl-glucosamine (Sigma-Aldrich, Merck Group, MO, USA). Standards were dissolved in ultra-pure water at three different concentrations (12, 25, and 50 mg/ml) to assure specificity of the peaks. A 20 µl liquid droplet was placed on a stainless-steel slide for each concentration of standard. Measurements were taken in duplicate for each concentration in the non-dried form with a Raman spectrophotometer inVia Qontor (Renishaw, Gloucestershire, UK). All spectra were recorded between 400 and 1800 cm^− 1^ wavenumber range (1 cm^− 1^ spectral resolution) with a 50x objective and a 785 nm (Near infrared) laser. An integration time of 10 s was used at 50% (approximately, 55 mW) laser power at the sample surface. The baseline was automatically selected and subtracted, cosmic rays were removed and a 5th order polynomial smoothing (Slavitzky-Golay) applied with the WiRE software (Renishaw, Gloucestershire, UK). Calibration using the 520 cm^− 1^ peak of a silicon wafer was performed before sample spectrum acquisition. All spectra obtained were then averaged for each glycan. Only the highest peaks (reaching at least 100 intensity counts) were selected for inclusion in the database.

### Tissue and serum Raman spectra acquisitions

For tissue acquisitions, FFPE stainless-steel slides were dewaxed by immersion in xylene for 18 h. Then the sections were rehydrated in decreasing concentrations of ethanol before being rinsed in distilled water and left to air-dry for at least 30 min using a modified protocol for Raman spectroscopy to minimize wax, xylene, and ethanol contamination from the samples [23]. For Raman measurements, 25 single spectra per section were randomly sampled in the region of interest within neoplastic tissue (750 acquisitions in total). To ensure optimum laser focus between each measurement, the samples were manually focussed. To ensure that neoplastic regions, rather than surrounding healthy tissue, were targeted, comparison with a corresponding immunohistochemically stained section with representative markers, EGFR (Abcam, Ab52894) and GFAP (Sigma, G3893) was used for reference. Offset spectra (repeats presenting obvious deviation from the characteristic signature) were removed from the dataset and the mean spectrum for each group was generated by calculating the arithmetic mean at each point. In total, more than 600 acquisitions were used in the tissue analysis (n = 198 for grade II, 196 for grade III, and 208 for grade IV).

Identical processing parameters were used for the serum samples. For blood serum acquisitions, a 20 µl drop was dispensed on the steel slide and directly processed while liquid. The same parameters were used as described above. Spectra were acquired from 5 random locations in the serum sample and the mean spectrum for each group was generated. In total, approximately 150 acquisitions were used in the serum analysis (n = 49 for control, n = 49 for grade III, and n = 50 for grade IV).

### Raman spectra acquisitions on cell culture monolayers and spheroid formation

Glioma cell lines A-172 (CRL-1620), SW1088 (HTB-12), and T98G (CRL-1690) were obtained and grown according to the American Type Culture Collection (ATCC) (Manassas, Virginia, USA) standard protocols. A-172 and T98G cell lines had been derived from GBM patients and SW1088 cell line from a grade III astrocytoma patient. The three cell lines used in this study were received from ATCC and certified by short tandem repeat DNA profiling authentication and a negative test for mycoplasma contamination.

Briefly, for monolayer culture, 1 × 10^5^ cells were seeded in a Petri dish containing a stainless-steel slide covered with 10 ml of ATCC-formulated DMEM medium, supplemented with 10% fetal bovine serum (FBS) and 1× antibiotic-antimycotic (Gibco, ThermoFisher Scientific). After 48 h of incubation (37 °C, 5% CO_2_), cell viability was checked via morphological changes under the microscope, and the slide was washed with PBS. Acquisitions on the cell monolayers were performed while the cells were still viable, and the slide was kept wet following PBS addition. One cell was targeted for each acquisition. In total, for monolayers, 12 cells were sampled for grade III and 28 for grade IV (n = 9 for A-172, and n = 19 for T98G).

For the 3D cell culture, spheroids were generated using the ‘hanging drop’ method, as previously described [24]. Briefly, 20 µl drops of cell suspension (20 × 10^3^ cells per ml) were applied to the inside of the petri dish lid so the drops hung upside down in a closed dish, filled with growth medium to prevent evaporation. After 48 h, the spheroids were collected with a pipette and washed once with PBS. For Raman acquisitions, spheroids were left in a small volume of PBS and dispensed inside a stainless-steel culture plate. One spheroid was targeted for each acquisition with 12 spheroids interrogated for grade III and 32 for grade IV (n = 10 for A-172, and n = 22 for T98G).

### Statistical analysis

The intensity values were rescaled between 0 and 1 using the min-max formula. Principal component analysis (PCA) was performed to reduce the dimensionality of the dataset and 2D/3D plots were generated for averaged and rescaled tissue and serum samples by exporting the dataset matrix into MATLAB R2021b (The Mathworks, Inc., Massachusetts). The entire spectral range between 400 and 1800 cm^− 1^ was used for PCA. The built-in classification learner application was used in MATLAB to generate the accuracy rates with PCA. The three largest principal components were used for the classification. For all data, 5-fold cross-validation was used. To calculate the significance of the difference observed between two grades at different manually selected peaks, a two-tailed unpaired t-test was performed with GraphPad Prism version 9.3 (GraphPad Prism software, California) on averaged rescaled intensities.

## Results

### Generation of an in-house glycosylation standards database

**Fig. 1:**
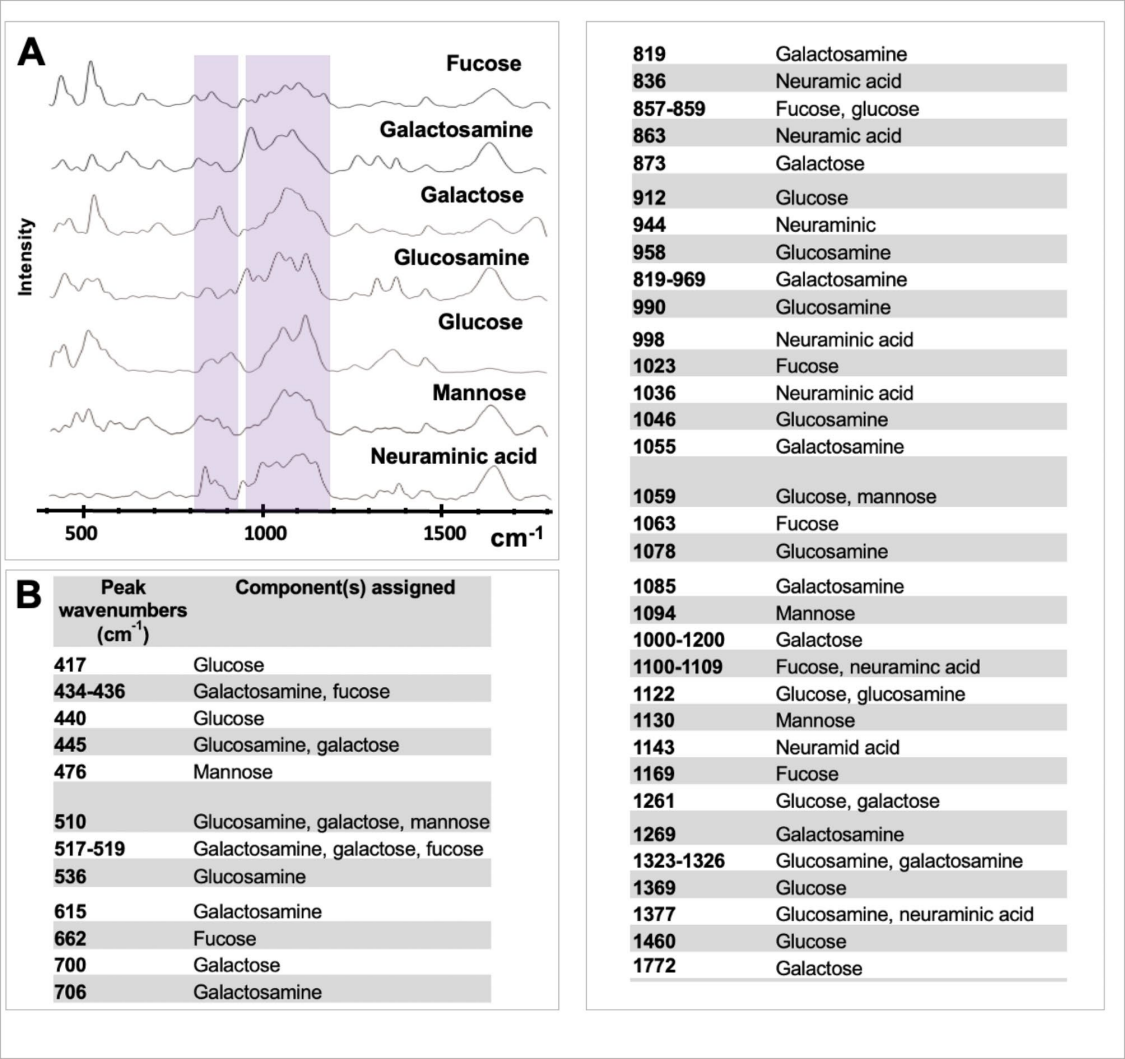
**Glycosylation database. (A)** Mean spectra for each glycan. Pink areas correspond to shared increased intensities between all glycans. **(B)** Characteristic peaks of the different glycans are listed

First, an in-house glycosylation database was generated for monitoring changes in glycosylation patterns in the samples. Seven glycan motifs most frequently present on heavily glycosylated proteins were included [25, 26]: glucose, fucose, galactosamine, galactose, glucosamine, mannose, and neuraminic acid (Fig. 1). The mean spectra for each glycan are represented in Fig. 1A and the characteristic peaks in Fig. 1B (note that the 1660 cm^− 1^ peak corresponds to the water peak, which did not change with the glycan concentration). The glucose spectra signature was in accordance with the reference spectrum from previous studies [27]. Glycan signatures shared common increased intensities, especially in the 800–910 cm^− 1^ and 950–1200 cm^− 1^ (Fig. 1A, pink areas), but also in the 400–600 cm^− 1^ (except for neuraminic acid), and the 1600–1700 cm^− 1^ regions (except for glucose) (Fig. 1A).

### Grade discrimination in glioma FFPE tissue samples using combined Raman spectroscopy and machine learning

**Fig. 2:**
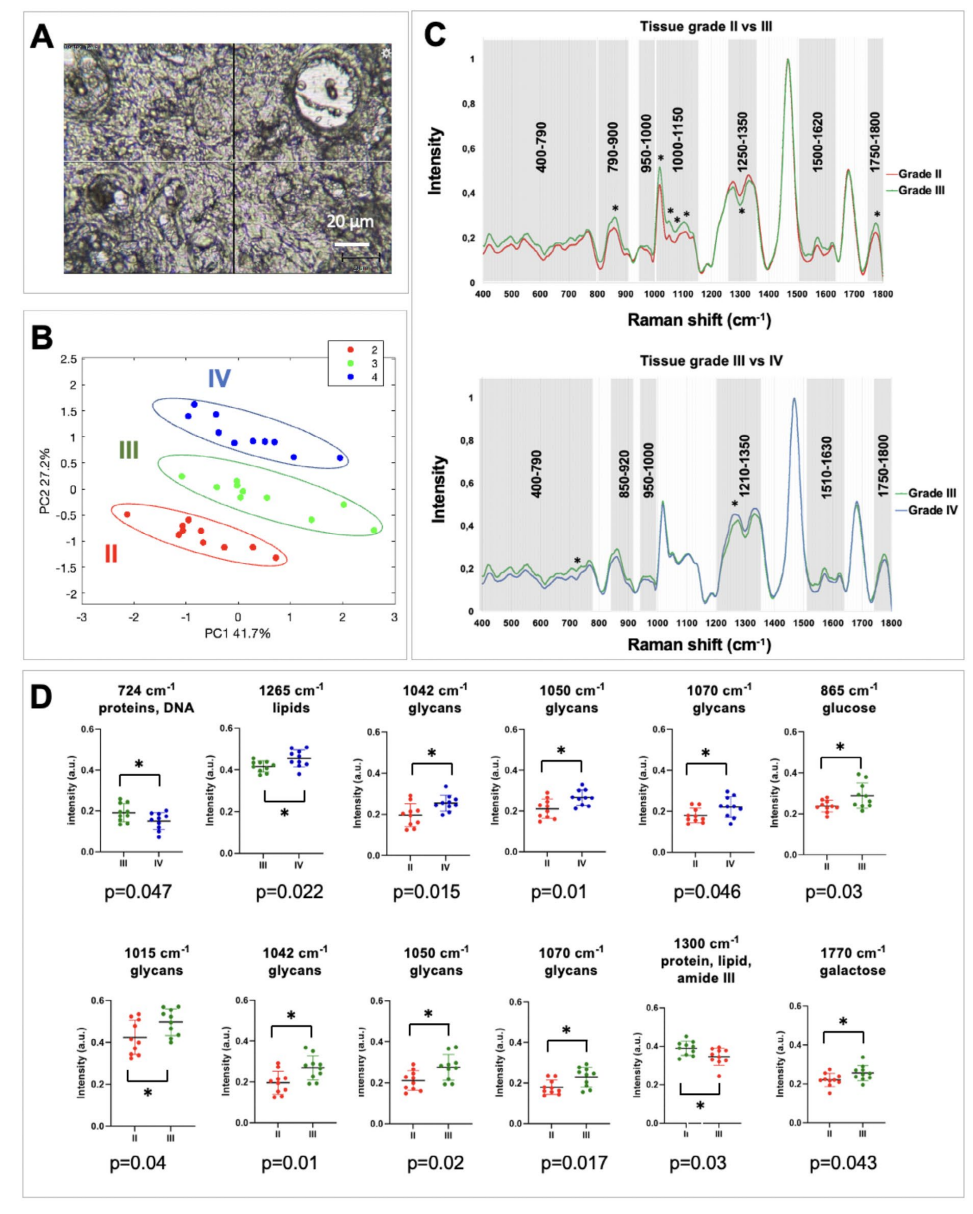
**Grade discrimination from FFPE tissue samples. (A)** Example view of a GBM tissue sample with the Raman microscope after dewaxing, before acquisition. Main structures are visible (vessels, red blood cells, cancer cells) and can be targeted. **(B)** PCA plot of the 30 glioma FFPE samples. Grade II (in red), grade III (in green), and grade IV (in blue) are easily discriminated by using the two largest principal components. **(C)** Pair-wise comparison between the averaged spectra of grade II and grade III, and grade III and grade IV. Asterisks indicate the peaks that were significantly different using an unpaired t-test. **(D)** Scatter plots of individual intensities (and mean ± standard deviation) at peaks showing significant difference using a two-tailed unpaired t-test. Circles drawn on PCA plot highlight trends assessed subjectively by eye

During RS interrogation on grade II, III, and IV glioma tissue (astrocytic only), visualization of the tissue structures was possible under the microscope after dewaxing (Fig. 2A). Non-relevant structures, such as vessels, abundant in grade IV glioma, were avoided with the help of the stained parallel slides (Fig. S1, Supplementary file). An averaged spectrum was generated for each patient after standard processing and rescaling. The variance and standard deviation between the patients were low for all the grades (an example for grade IV is shown in Fig. S2, Supplementary file).

PCA and classification were performed to evaluate whether the main variations between the samples could be assigned to their histological grades. The 2D PCA plot using the two largest PCs is represented in Fig. 2B for the three grades (II-IV). The two largest components, PC1 and PC2, were able to clearly discriminate all the patients in accordance with their grades and explained together 69% of all the variance between the samples (Table S2, Supplementary file for the percentage explained for all PCs). We then used classification learning to calculate the accuracy rate of this discrimination by using a 5-fold cross-validation on different model types and with the three largest PCs. Linear Support Vector Machine (SVM) provided the highest accuracy rate to discriminate between the grades in the tissue: 80% between grade II and III, 85% between III and IV, and 75% between II and IV (Table 1). These results suggest that RS, combined with PCA and machine learning, predicts the histological grade of astrocytic tumour tissue samples with very good accuracy from dewaxed tissue samples.

**Table 1:** **Classification accuracies.** Results from machine learning for six different classification model types when using the first 3 largest PCs from the PCA and a 5-fold cross-validation, between all groups for tissue and non-dried (fresh) serum. SVM = Support Vector Machine, KNN = k-nearest neighbors algorithm

Classification accuracy (%)	Linear SVM	Linear discriminant	Cosine KNN	Logistic regression	Bilayed neural network	Narrow neural network
**Tissue**						
II vs. III	80%	75%	70%	70%	40%	50%
II vs. IV	75%	70%	60%	70%	45%	55%
III vs. IV	85%	60%	75%	60%	55%	50%
All grades	63.3%	60%	53.3%	x	36.7%	30%
**Fresh serum**						
CTRL vs. III	75%	75%	80%	85%	90%	80%
CTRL vs. IV	60%	65%	70%	85%	85%	85%
III vs. IV	60%	65%	65%	75%	90%	85%
All grades	56.7	53.3%	60%	x	63.3%	66.7%

Since IDH1 genotype is crucial in the current diagnostic decisions, the discrimination between wild type and mutated IDH1 was tested. As expected, – since IDH genotype is strongly correlated to the histology – the two genotypes could be easily discriminated on the PCA plot, like the histological grades. The three first PCs explained 76% of the variance between the samples and the accuracy rate was 80% (Linear SVM) (Fig. S3, Supplementary file). The wild-type group, which is strongly associated with higher histological grades, was slightly more dispersed on the PCA plot. The mutated group, strongly associated with lower glioma grades and presenting better prognosis, displayed less variance between the samples.

### Analysis of the biomolecular changes in glioma FFPE tissue grades

Pair-wise comparison of the representative spectra was then conducted between each grade to identify differences that could discriminate the grades. Comparison of grades II and III and grades III and IV are represented in Fig. 2C. To assign peaks showing variation between the two grades to specific biomolecules, the in-house glycosylation database and a literature-based summary were used [27–36] (Table S3, Supplementary file).

Overall, the Raman peaks of grade III dominated in intensity compared with grade II (Fig. 2C, grade II vs. grade III) and were largely assigned to different glycans (see Table 2 for the detailed assignments). The most important difference was localized within the 1000–1150 cm^− 1^ region, which corresponded to the high-intensity regions shared by all glycans (Figs. 1 and 2C). Assignments to other biomolecules present in several spectral areas could also be made from the literature database (Table 2): cholesterol, proteins, haemoglobin, DNA, GAG, collagen, proteins, lipids, and phospholipids, indicating differences between grade II and III.

**Table 2:** **Summary of changes observed in tissue and serum and their tentative assignments**. Assignments were made from the glycosylation database and the literature-based general database [27, 29–36, 65, 66]. NA = neuraminic acid

Tissue observations (cm^− 1^)		Tentative assignments
**II < III**	400–790	All glycans except NA
	790–900	Galactosamine, galactose, fucose, NA, glucose
	950–1000	Glucosamine, galactosamine, NA, fucose
	1000–1150	All glycans
	1750–1800	Galactose
	859	Glucose (843)
	912	Glucose (910–911)
	1094	Mannose (1095)
	400–780	Cholesterol and proteins (446–476, 700–703), proteins (500–550, 636–646), DNA (498, 676), proteins and DNA (725–729), haemoglobin (670), DNA, proteins, and haemoglobin (743–790)
	790–900	Collagen (818), proteins (823, 880–890), proteins, collagen, and GAG (857)
	950–1000	Proteins (959, 1003)
	1000–1150	Proteins (1032, 1127), lipids (1064–1068, 1129), phospholipids and collagen (1074),
	1500–1620	DNA, proteins, haemoglobin (1573–1585), proteins (1602–1607)
**III < II**	1250–1360	Amide III (1230–1306), cytochrome C (1358), lipids (1263), phospholipids (1313), collagen (1323)
**IV < III**	400–790	All glycans except NA
	850–920	Fucose, glucose, NA, galactose
	950–1000	Glucosamine, galactosamine, NA, fucose
	1750–1800	Galactose
	1510–1550	Carotenoids (1521)
**III < IV**	1210–1350	Haemoglobin (1225), GAG (1242), proteins, collagen, lipids (1230–1306), lipids (1263), phospholipids (1313), collagen, proteins (1322), DNA, proteins (1331–1338)
**Serum observations** **(cm** ^**− 1**^ **)**		**Tentative assignments**
**III > CTRL**	400–610	Galactose, mannose, fucose, glucose, galactosamine
	680–860	All glycans
	1010–1120	
	1290–1340	Glucose, NA, glucosamine, galactosamine
	1700–1750	Lipids (1732)
**III < CTRL**	1420–1470	Phospholipids (1441–1445)
	1610–1660	Lipids (1654)
**III > IV**	420–600	All glycans except NA
	680–860	Galactose, galactosamine, NA, fucose, glucose
	1000–1130	All glycans
	1210–1340	Glucose, galactose, glucosamine, glactosamine
	450–500	Cholesterols (446–476, 700–703)
	689–750	
	1010–1070	Lipids (1064–1068, 1220–1306, 1400, 1732)
	1290–1340	
	1380	
	1700–1750	
	1145–1160	Carotenoids (1157, 1521)
	1500–1520	
**III < IV**	880–920	Glucose (910–911), proteins (880–890)

Changes in intensities between the malignant grades III and IV were smaller and the 1000–1200 cm^− 1^ region showed no difference (Fig. 2C, grade III vs. IV). Overall, there was a slight decrease in grade IV compared with grade III and most spectral ranges that differed could be assigned to glycans from the glycosylation database but also to the carotenoid motif from the literature summary (Table 2). The increase of intensity in the 1210–1350 cm^− 1^ in grade IV, compared to grade III, could also be interpreted as an increase in collagen, proteins, lipids, haemoglobin, GAG, and DNA (Table 2).

Comparison between grade II and grade IV spectra showed an increase in grade IV compared with grade II in the glycan-riche region 1000–1150 cm^− 1^ region (data not shown). According to the literature, this region could also be assigned to an increase in proteins (1032 cm^− 1^), lipids (1063 cm^− 1^), phospholipids and collagen (1074 cm^− 1^), whereas all the other regions had very similar intensities (data not shown).

To further assess the significance of these changes observed between the different grades and to screen for specific individual peaks that could be useful for diagnosis, we selected the peaks that showed the largest intensity difference between the two grades. t-tests were performed for those peaks, meaning, intensity values for each specific peak were compared between the two grades (Fig. 2D, significance is indicated by asterisks in Fig. 2C). Differences between grades II and III were overall more significant than differences between grade III and IV and notably, the 1000–1150 cm^− 1^ region, assigned to glycans, showed significant difference for the four peaks selected (1015, 1042, 1050, 1070 cm^− 1^). These peaks could not be assigned to any other component than glucose, according to the literature database. In addition, other peaks that showed significant difference could be assigned to various well-characterized biomolecules (Fig. 2D).

The PCA loadings plot was next generated to visualize the contribution of each of the largest PCs to the variances observed between the grades across the spectrum. In addition to the t-tests performed, this type of representation can further confirm what areas of the spectrum contribute the most to the significant differences between the grades. The results are shown in Figure S4. The different PCs are usually widely distributed throughout the variables.However, the plot clearly showed that two regions had major contributions in the grade discrimination, namely, the 950–1200 cm^-1^ and the 1250–1350 cm^-1^ regions of the spectrum, which further confirmed the importance of the glycosylation changes discussed earlier but also the relevance of this method.

Finally, the Raman signatures of IDH1-mutated and IDH1-wild-type patients were compared. The comparison between the two signatures looked very similar to the comparison between grade II (containing almost only IDH1-mutated patients) and grade IV (containing almost only IDH1-wild type patients); the wild-type signature showed mainly an increase in the 1000–1150 cm^− 1^ (characteristic of glycans) in comparison with the IDH1-mutated signature (Fig. S5, Supplementary file). This implies that the level of overall glycosylation seemed to account widely for the high accuracy of Raman discrimination between IDH1-mutated and IDH1-wild type patients.

### Grade discrimination from blood serum samples using combined Raman spectroscopy and machine learning

Next, we wanted to investigate whether discrimination between the most challenging glioma grades, grade III and IV, could also be achieved from liquid biopsies as they are collected less invasively. Raman spectra were acquired from blood serum samples on stainless steel slides. A control group of non-glioma benign samples was included. The inter-patient standard deviation was higher (Fig. S6, Supplementary file) than previously observed in the tissue samples. This may indicate that RS can detect more subtle biological changes from liquid serum than from dewaxed fixed tissue, which could be explained by the nature of this type of samples (non-paraffinized and in their native state).

**Fig. 3:**
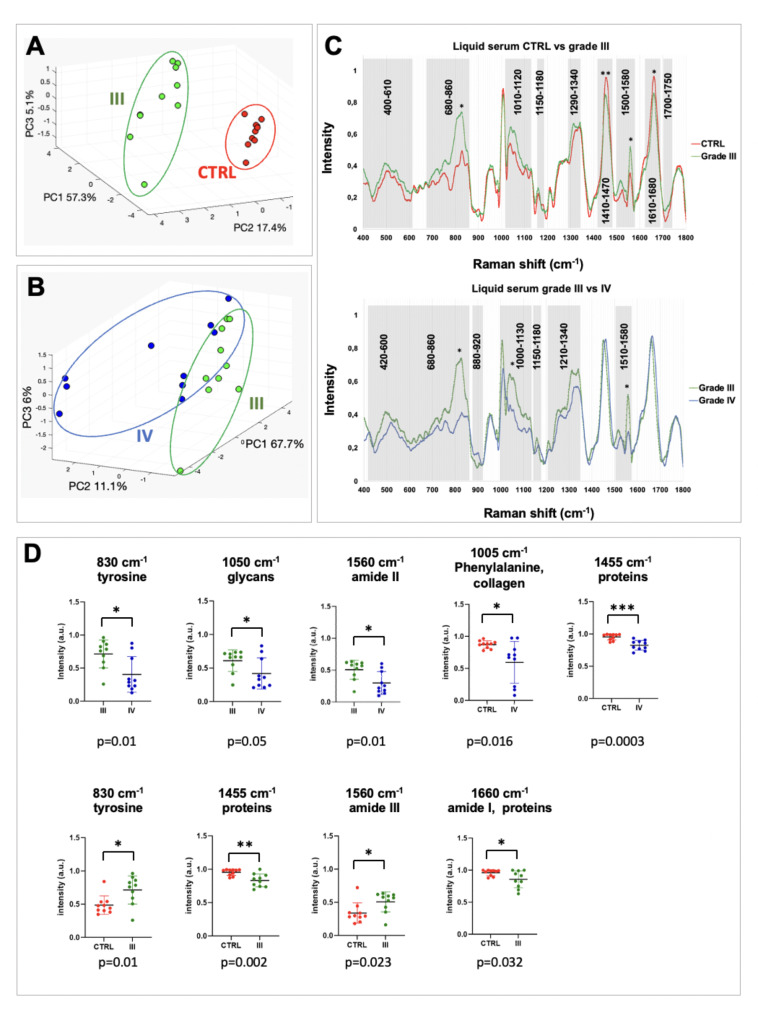
**Grade discrimination from fresh serum samples.** 3D PCA plot of glioma blood serum samples using the three largest principal components (PC1, PC2, PC3). Control (CTRL) and grade III samples can be discriminated **(A)** as well as grade III and grade IV **(B)**, grade III and grade IV have a wider distribution in comparison with control samples. **(C)** Pair-wise comparison between the averaged spectra of control and grade III, and grade III and grade IV. Asterisks indicate the peaks that were significantly different using the t-test, while areas shaded in grey highlight important differences. **(D)** Scatter plots of individual intensities (and mean ± standard deviation) at peaks showing significant difference using a two-tailed unpaired t-test. Circles drawn on PCA plot highlight trends assessed subjectively by eye

The 3D PCA plot of the control and grade III groups using the three largest PCs was produced (Fig. 3A). The three PCs represented together 80% of all the variance. The discrimination between control and grade III patient serum samples was apparent and with no overlap and control samples showed relative intragroup spectral homogeneity, whereas grade III patients showed more dispersion and intragroup heterogeneity (Fig. 3A). The 3D PCA plot of grade III and grade IV groups using the first PCs is also represented in Fig. 3B. The three largest PCs represented together 85% of all the variance between the samples. Grade IV samples displayed a moderate increase in distribution size compared with grade III samples (Fig. 3B).

The neural network models gave the best discrimination rate for serum with accuracy rates of 90% between control and grade III, 85% between control and grade IV, and most importantly 90% between grade III and IV (Table 1).

Finally, the discrimination between the two IDH1 genotypes was tested as previously with the tissue. The three first largest PCs explained 84.5% of the variance between the samples and the accuracy rate was overall lower than for the IDH1 discrimination in tissue (65% with Linear SVM) and the clusterization was visibly more challenging to make on the PCA plot than previously with the tissue. However, a clear trend of clusterization could be observed (Fig. S7, Supplementary file) and the logistic regression gave an accuracy of 85%, which suggests that a small drop of serum could represent a non-invasive and fast method to help IDH diagnostics in clinics.

### Analysis of the biomolecular changes in glioma serum samples

A pair-wise comparison of the representative spectra was further conducted to identify the biomolecular changes between glioma grades and control in fresh serum samples (Fig. 3C). Grade III patients showed dominant intensities in Raman shift compared to controls, mostly assigned to glycans (Table 2). A decrease observed in grade III patients could be assigned to phospholipids and lipids. A similar trend was observed between grade III and grade IV gliomas. Grade III samples showed increased intensity levels compared to grade IV in regions that we previously assigned to glycans. These regions have also been assigned to cholesterol and lipids from the literature database. Moreover, regions assigned to carotenoids showed decreased intensity levels in grade IV compared with grade III (Table 2). Interestingly, Raman peaks assigned to glycosylation changes in serum, increased in intensity in grade III compared to non-glioma benign tumours (control), but decreased between malignant grades (grade III vs. IV), which aligns with the tissue findings.

Peaks that showed significance are represented in Fig. 3C-D. The large difference observed within the 700–850 and 1000–1130 cm^− 1^ wavenumber regions assigned to glycans was significant between controls and grade III, between grade III and grade IV samples in the 700–850 cm^− 1^, and between grade III and grade IV samples in the 1000–1130 cm^− 1^ region. The 1455 cm^− 1^ peak was the most significant for discrimination between controls and grade IV (p = 0.0003) and controls and grade III (p = 0.002) samples. In total, three well-characterised peaks could be used to significantly discriminate grade III and grade IV (830 cm^− 1^, p = 0.01; 1050 cm^− 1^, p = 0.05; 1560 cm^− 1^, p = 0.01, respectively assigned to tyrosine, glycan, and amide III) (Fig. 3D).

### Grade discrimination from cell lines grown in monolayers and spheroids

Next, we investigated whether the changes observed between grade III and grade IV tissue glioma samples would also be reflected at the cellular level using different cellular models (2D-monolayers and 3D-spheroids) in vitro. This would allow assessment of whether grade III and grade IV cell lines could be discriminated and, further, whether there is a difference in spectra between single cells and multicellular spheroids. Unlike FFPE tissue samples, live cells were not treated with numerous preservation and processing steps, involving chemicals, meaning the integrity of the lipid content is potentially more preserved in cell lines than dewaxed tissue [37]. In addition, it was important to confirm that the discrimination between glioma high grades can be repeated at the cellular level for different applications and to further study the change in composition.

**Fig. 4:**
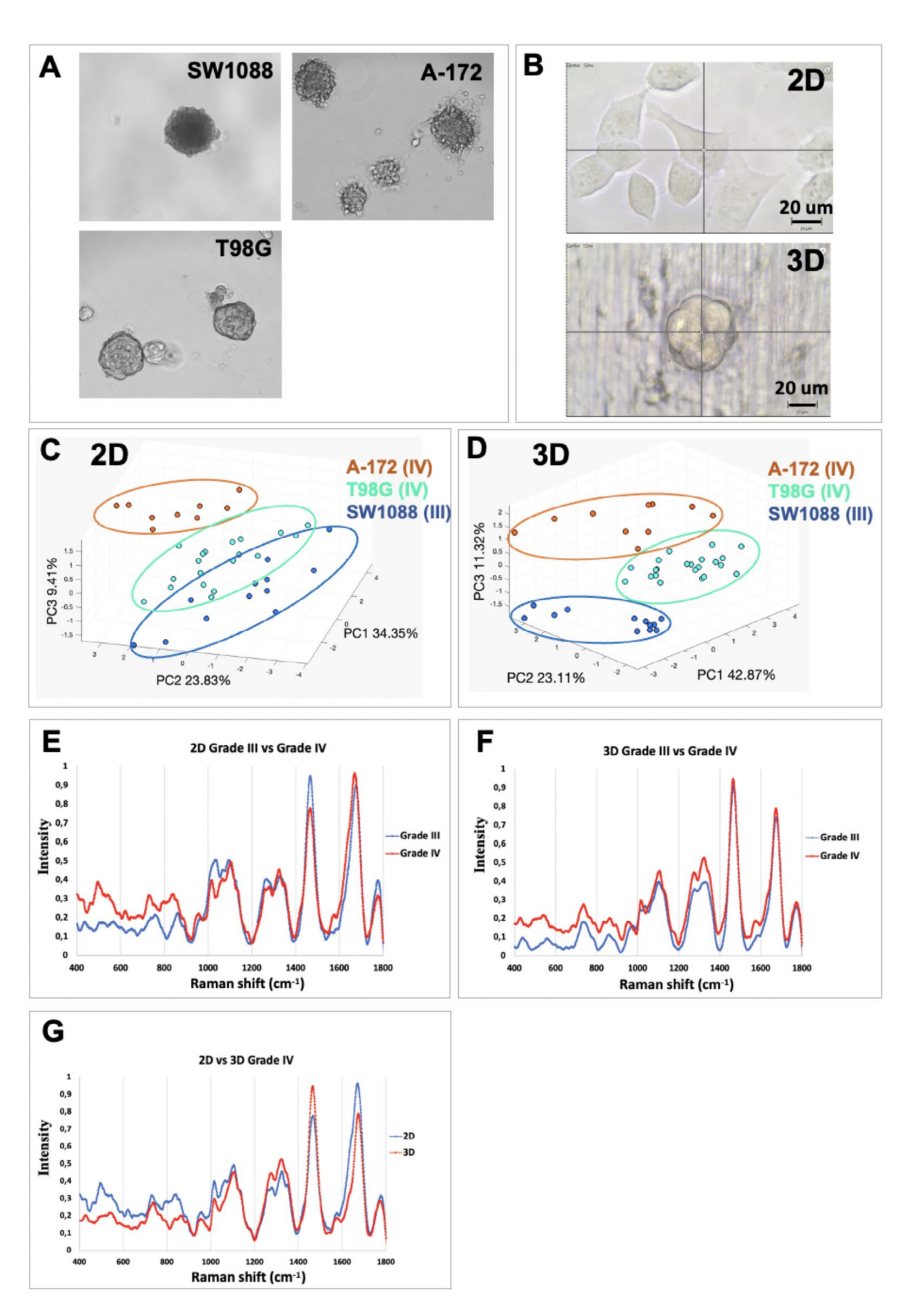
**Discrimination in glioma cell lines. (A)** Images representing the multicellular spheroids generated by the hanging drop method for the three cell lines, magnification 200X. **(B)** Images representing cells grown in 2D (monolayers) and 3D (spheroids) under the Raman confocal microscope after 48 h before acquisition. **(C)** 3D PCA plot of the individual cells (one dot represents one cell) grown in 2D using the three first PCs. **(D)** 3D PCA plot of all the individual spheroids using the three largest PCs. **(E)** Pair-wise comparison between the mean spectra of grade III and grade IV individual cells. **(F)** Pair-wise comparison between the mean spectra of grade III and grade IV spheroids. **(G)** Pair-wise comparison between the mean spectra of grade IV 2D and grade IV 3D cells. Circles drawn on PCA plot highlight trends assessed subjectively by eye

Therefore, grade III (SW1088) and grade IV (GBM) (T98G and A-172) cell lines were grown in 2D monolayers and 3D spheroids in the same growth media and dispensed before acquisitions. Representative spheroids images for each cell line are shown (Fig. 4A). For each cellular model, one cell or spheroid was targeted with RS microscopy (Fig. 4B).

The cell lines grown in monolayers (Fig. 4C, E) or spheroids (Fig. 4D, F) were first compared using PCA. For single cells grown in monolayers, the three largest PCs explained together 67.6% of the variance between the samples. The discrimination rate between the cell lines was 85% when using a 5-fold cross-validation (linear discriminant analysis). For cells grown in 3D spheroids, the first three PCs explained 77.3% of the variance and the discrimination rate between the cell lines was 68.2%.

### Spectral differences between cellular models and biomolecular signatures

The mean spectra of grade III and grade IV cells were compared for both monolayers (Fig. 4E) and spheroid methods (Fig. 4F). Between 400 and 900 cm^− 1^, grade IV cells displayed higher overall intensity for both methods. The higher intensity observed in grade IV (GBM) cells in this region could reflect a higher level of protein content since this region is widely associated with proteins as well as DNA (Table 2).

For monolayers, within a region between 1000 and 1100 cm^− 1^ previously largely assigned to glycans, a decrease in intensity was observed in grade IV compared to grade III, as well as in the 1760–1775 cm^− 1^ region, assigned to galactose. The decrease of glycans within the 400 and 900 cm^− 1^ regions observed between grade III and grade IV tissue was not observed at the cellular level. Targeting the spheroids, the signature was very similar between 1000 and 1800 cm^− 1^, with an increase in the 1200–1400 cm^− 1^ region, which could be assigned to the amide III vibration of proteins, lipids, and DNA. The grade IV signatures from single cell or multicellular spheroids were also compared (Fig. 4G). The intensity given by the 3D spheroids was lower between 400 and 1200 cm^− 1^ in regions largely assigned to proteins and glycans, while the intensity was higher in the 1200–1400 cm^− 1^ region, corresponding to lipids, DNA, and amide III. As expected, targeting a single cell or a multicellular spheroid does not provide the same biomolecular signature reflecting their differences in cellular metabolism and other biochemical pathways.

## Discussion

Raman spectroscopy (RS) presents many advantages to be used as a diagnostic tool, including non-demanding sample preparation [38, 39]. In this study, combining RS with machine learning, changes in the biomolecular composition of glioma tumour tissue and serum samples were detected, with a focus on glycosylation patterns. Firstly, our approach was used to discriminate grade II, III, and IV glioma from FFPE tissue samples, which are systematically available as stored archival samples. RS could accurately classify glioma patients (Fig. 2) according to their histological grades. Importantly, the two consecutive malignant glioma grades (III and IV), were discriminated with a high degree of accuracy of 85% (Fig. 2). Moreover, Raman spectra differences were statistically significant between the grades (Fig. 2C-D) on selected individual spectra, implying this method is objective and may help diagnostics in clinics. RS was also able to predict with good accuracy the IDH1 genotype of the patients, which is the marker used currently for cytogenetic classification [40]. This result was expected since histological features and the IDH1 genotype are strongly correlated. The results suggest that RS interrogation of FFPE slides, that are available in clinics, could help to confirm histology and IDH1 genotyping. With further development, this diagnostic method could prove more efficient, faster and less expensive, but also interpretable with more objectivity in comparison with immunostaining methods. Moreover, the averaged spectral difference between the two IDH1 genotypes was shown to be clearly condensed in the glycosylation region. This implies that the assessment of only a restrained spectral range would be enough for diagnosis, which would reduce the diagnostic time.

Comparing the Raman spectra in tissue from grade II and III patients, changes of intensity were assigned to an increase in crucial biomolecules, identified as proteins, haemoglobin, DNA, collagen, and lipids (Fig. 2C). Proteins, haemoglobin, and DNA have been shown to increase during cancer progression due to higher cell density and vascular proliferation, which are also typical histological characteristics of GBM [32, 41]. In addition to collagen rearrangement, a switch in the level of the different collagen types, has also been shown to occur during glioma progression [29, 42–44]. Overall, our study confirmed that Raman peaks that increase during glioma progression, could be assigned to an increase in collagen, proteins, lipids, haemoglobin, and DNA levels [12, 32, 45]. In addition, there was a decrease in antioxidant carotenoids in grade IV compared to III, a common feature in RS cancer research studies [12, 46, 47]. It has been suggested that the peak intensity reduction assigned to carotenoids on Raman spectra in GBM could be used as a biomarker in brain cancer to assess tumour aggressiveness [12].

Importantly, an increase in the intensity of the biomolecular signature could largely be assigned to glycans (between grade II and III tissue samples, Table 2; Figs. 1 and 2C). Of note, FFPE samples have been shown to preserve almost unaltered N-glycan signature after the deparaffinization procedure [48]. As expected, the differences between grades III and IV were smaller (Fig. 3C). When analysing the spectral ranges assigned to glycosylation, grade III patterns were higher compared to grade IV. An important number of glycans have been found to be upregulated in grade III compared to grade II and IV [49]. In addition, different glycans have been found in their truncated version in grade IV glioma [50], which implies that the total level of glycans could be lower in this type of cancer. This is in accordance with the highly altered glycan biosynthesis observed during cancer progression that accounts for the phenotypic aggressiveness [51]. Our study included glycans typically found on glycosylated proteins [22, 26]. Importantly, such altered glycosylation during glioma progression has been shown to contribute to the immunosuppressive nature of the glioma microenvironment [50], indicating the importance of those changes in immunotherapeutic treatments. Overall, the total glycosylation pattern follows complex dynamics during transformation, captured by RS, and might be exploited for glioma monitoring and classification.

Liquid biopsies from patient serum samples feature significant advantages in glioma diagnosis: they are minimally invasive, can be collected at multiple times for disease and therapeutic monitoring, and are considered more homogeneous than a complex tissue environment, especially when used in their native liquid state, which is applicable since water has a low influence on RS [9]. Therefore, from a technical viewpoint, the use of serum instead of solid biopsies presents several advantages. For instance, in the present study, the RS acquisition time was reduced by a factor of five (12–15 min per patient) in comparison with the tissue, due to the homogeneity of liquid samples. The accuracy rate was slightly better in serum than tissue (85%) (Table 1); importantly, grade III and grade IV patients were discriminated with an accuracy of 90% in serum. RS was also able to discriminate again, with good accuracy, the two IDH1 genotypes from fresh serum. Like tissue, this bolsters the use of RS in the clinics, with serum representing a less invasive collection method for the patients. IDH genotyping from blood serum with Raman spectroscopy would represent a fast, non-invasive, objective, and cost-effective diagnostic strategy to help both histological grading and IDH genotyping, in parallel with monitoring glycosylation and other biological changes occurring during cancer progression − a holy grail for liquid biopsy cancer detection.

Interestingly, significant heterogeneity was observed within the high-grade glioma samples when using serum, reflecting the tumour heterogeneity; while, as expected, the control group showed a much smaller distribution on the PCA plot (Fig. 3). Inter-patient heterogeneity in the blood is expected to increase in cancer patients and has been described in previous RS studies, notably in nasopharyngeal cancer [30]. This suggests that biological changes occurring during cancer progression are patient-specific, and that this specificity is reflected in serum, which may be further exploited for personalized medicine [52]. Among other biomolecular changes, the level of carotenoids was again reduced in the serum of grade IV, compared with grade III glioma patients like other cancer types such as cervical cancer, meningioma, and breast cancer [47, 53, 54]. This shows that a high level of circulating carotenoids, which have antioxidant properties, could play a protective role against malignant tumours, including GBM.

The same glycosylation trend was observed in tissue and serum, with higher glycosylation in grade III compared to grade IV. This result suggests a promising use of glycosylation signatures from liquid biopsies for cancer diagnostics and progression monitoring. The presence of individual glycoproteins in blood serum has been shown to be useful for cancer diagnostics [22, 55]. Glycoproteins, such as mucins, are in their aberrant glycosylated forms specifically in advanced cancer, meaning they could play a direct role in neoplasia and have been suggested as potential serum biomarkers [56–62].

Further studies on a large cohort of patients should be conducted in clinics, firstly to confirm the results of this study, but also to evaluate how RS aligned with even more precise sub-classifications of glioma, such as the distinction between astrocytoma and oligodendroglioma. The method presented here offers significant advantages over other technologies. In addition to cost benefits, RS combined with PCA is a reasonably simple and objective method that can be applied in clinics for both tumour classification and detection of biological changes. The approach followed here relies mainly on PCA, which has shown previous success in classifying cell behaviour even in the presence of a small sample size, being based on linear transformations [63]. Moreover, this method could allow monitoring at different time points thanks to its application on serum samples, collected in a non-invasive way. Non-spectroscopic methods and technologies to classify brain tumour samples have been suggested recently, including complex methylation array processing which can be used on solid biopsies [64]. This method may be less convenient in clinics at different time points; however, methylation classification is very precise, with many refined molecular clusters holding great promise for precision medicine. Future studies investigating whether different sub-molecular classes such as methylation, and others, such as the 1p19q correlation, could be discriminated with RS and aligned with spectroscopic analysis offering new, exciting potential diagnostic avenues.

Finally, we further studied the discrimination between grade III and grade IV glioma at the cellular level (Fig. 4). Different cell lines could be discriminated by PCA from single cells and spheroids with high accuracy (Fig. 4C and D), indicating that grade discrimination may be achievable on single live cells. Both methods gave similar but also different biomolecular signatures for GBM cells (Fig. 4E-G). The intensity given by the spheroids was decreased in the regions largely assigned to proteins and glycans, while the intensity was higher in regions, corresponding to lipids, DNA, and amide III (Fig. 4G). This result can be explained considering the complex tumour microenvironment of the tissue compared to the single cell and the multicellular spheroids. The increased level of lipid, DNA, and amide III band in the 3D-spheroid signature could reflect that several nuclei are targeted by RS but also a change of lipid and protein configuration due to cellular interactions. More importantly, targeting single cells with the proposed RS approach could provide an additional benefit to the current single-cell technologies in the era of cancer personalized medicine.

## Electronic supplementary material

Below is the link to the electronic supplementary material.

Supplementary Material 1

## Data Availability

The datasets generated and/or analysed during the current study are available from the corresponding author on reasonable request.

## List of abbreviations

GBM: Glioblastoma
RS: Raman spectroscopy
WHO: World Health Organization
FFPE: Formalin-fixed paraffin-embedded
PTM: Post-translational modifications
EGFR: Epidermal growth factor receptor
GFAP: Glial fibrillary acidic protein
PCA: Principal component analysis
PC: Principal component
GAG: Glycosaminoglycan
IDH: isocitrate dehydrogenase
