# Supplementary material for: Glycosylation spectral signatures for glioma grade discrimination using Raman spectroscopy
Source: BMC Cancer. 2023 Feb 21;23:174. doi: 10.1186/s12885-023-10588-w (PMC9942363; doi:10.1186/s12885-023-10588-w)
Supplement: Supplementary file 1 — Supplementary Material 1 [file 12885_2023_10588_MOESM1_ESM.docx]

**Supplementary File**

**Glycosylation Spectral Signatures for Glioma Grade Discrimination Using Raman Spectroscopy**

Agathe Quesnel^1,2^, Nathan Coles^1,2^, Claudio Angione,^2,3,4^ Priyanka Dey^1,2,5^, Tuomo M. Polvikoski^6^, Tiago F. Outeiro^6,7,8,9^, Meez Islam,^1,2^ Ahmad A. Khundakar^1,2,6^, Panagiota S. Filippou^1,2*^

1. School of Health & Life Sciences, Teesside University, Middlesbrough, TS1 3BX, United Kingdom
2. National Horizons Centre, Teesside University, 38 John Dixon Ln, Darlington, DL1 1HG, United Kingdom
3. School of Computing, Engineering & Digital Technologies, Teesside University, United Kingdom
4. Centre for Digital Innovation, Teesside University, United Kingdom
5. School of Pharmacy and Biomedical Sciences, University of Portsmouth, Portsmouth PO1 2UP, UK
6. Translational and Clinical Research Institute, Faculty of Medical Sciences, Newcastle University, Newcastle upon Tyne, United Kingdom
7. Department of Experimental Neurodegeneration, Center for Biostructural Imaging of Neurodegeneration, University Medical Center, Göttingen, Germany
8. Max Planck Institute for Multidisciplinary Sciences, Göttingen, Germany
9. Deutsches Zentrum für Neurodegenerative Erkrankungen (DZNE), Göttingen, Germany.

* Correspondence should be addressed to:

Dr. Panagiota S. Filippou

School of Health & Life Sciences,

Teesside University, Middlesbrough, TS1 3BX, UK

Tel: +44(0)1642-384631

E-mail: [P.Philippou@tees.ac.uk](mailto:P.Philippou@tees.ac.uk)

ORCID: 0000-0003-3974-988X


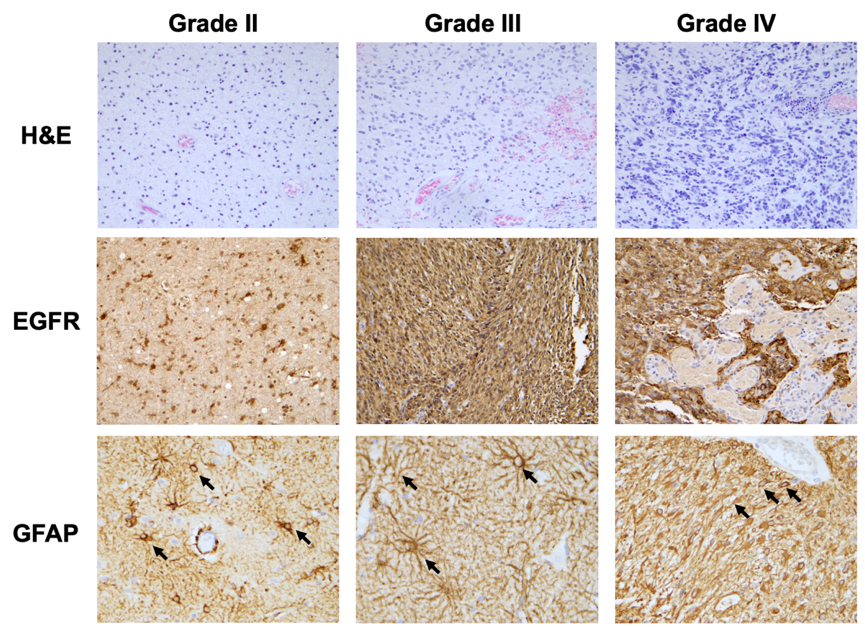


**Figure S1. Neoplastic region labelling in parallel slides.** Haematoxylin and eosin (H&E), EGFR (200X), and glial fibrillary acidic protein (GFAP) staining (400X) were performed on three parallel glass slides to help identify neoplastic regions. H&E staining reveals cellular density. The slides were also stained for EGFR (expressed in neoplastic cells in 90% of gliomas) and GFAP with the IHC method. Black arrows indicated expression of GFAP in glial cells, revealing their shape (highly altered in GBM).

**
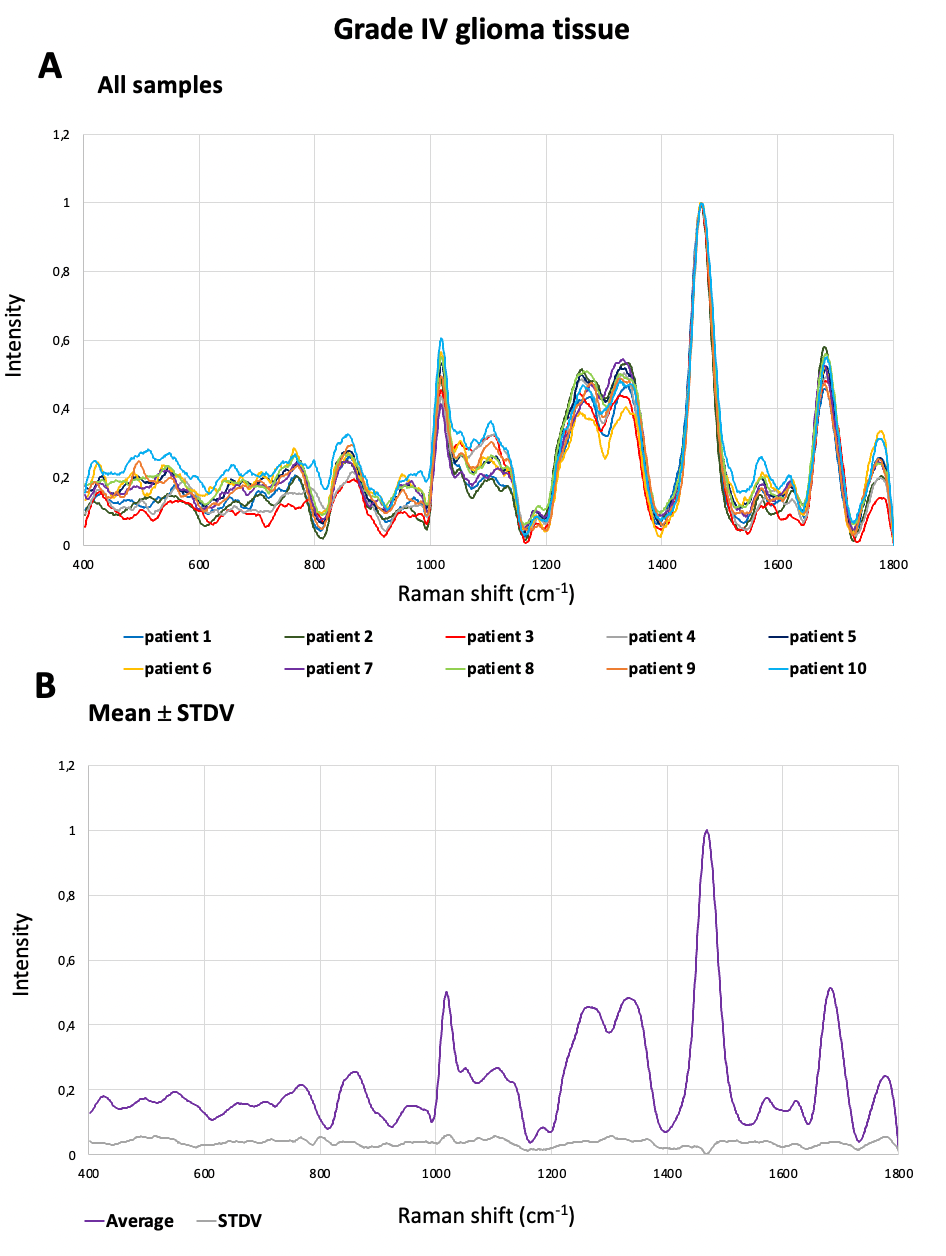
**

**Figure S2. Mean tissue spectra of grade IV glioma patients. (A)** All mean spectra for each patient (each sample). **(B)** Mean spectra (in purple) for grade IV with the standard deviation (in grey).

**
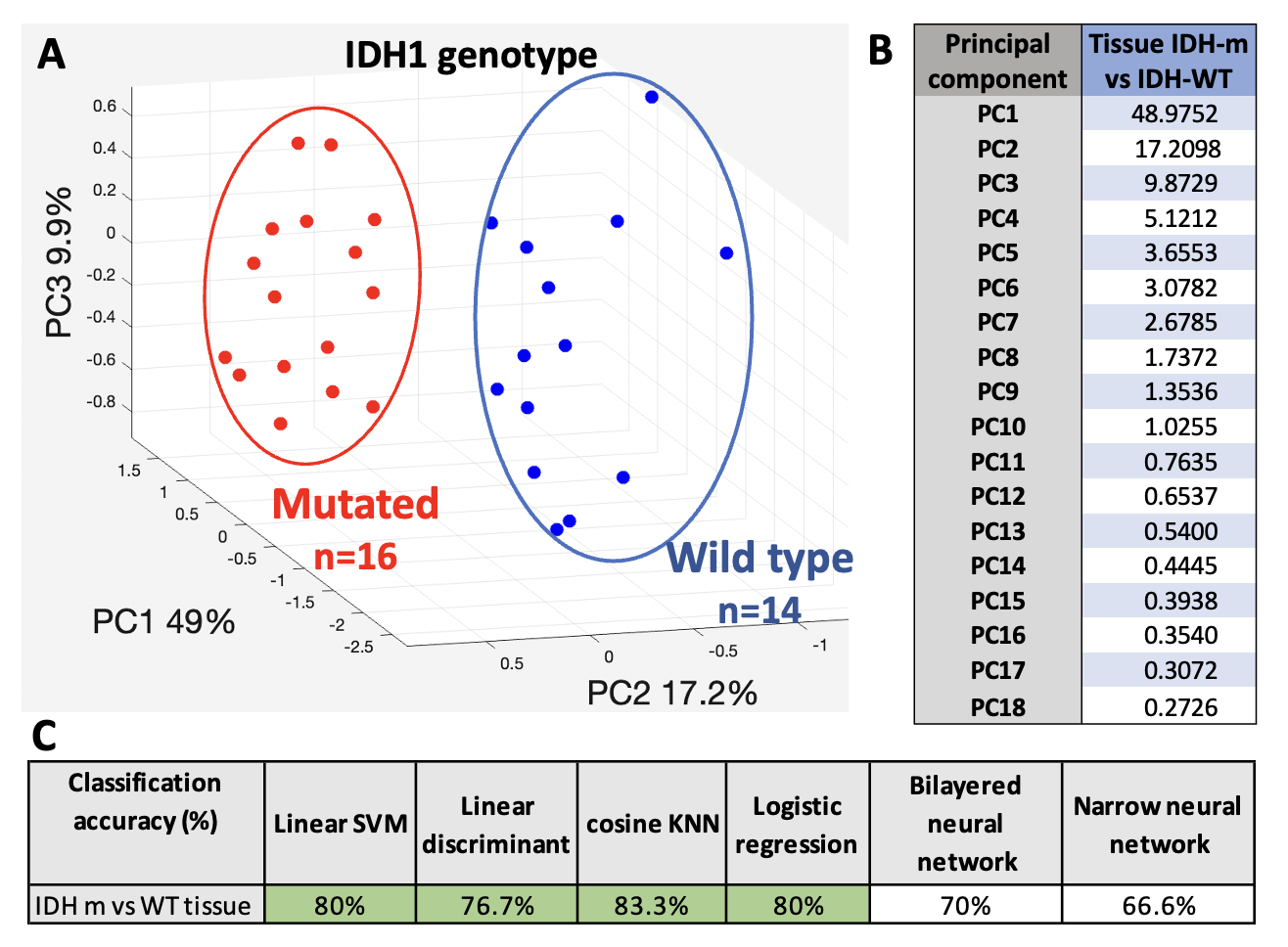
**

**Figure S3. Discrimination between IDH1-mutated and IDH1-wild type patients in tissue. (A)** 3D PCA plot of IDH1-mutated (n=16) and IDH1-wild type (n=14) patients (grade II, III and IV). The circles drawn on the plot represent trend determined subjectively by eye. **(B)** Percentages of variance explained by each PC. **(C).** Classification accuracy for different models of classification learning. Accuracy rate above 75% are highlighted in green.


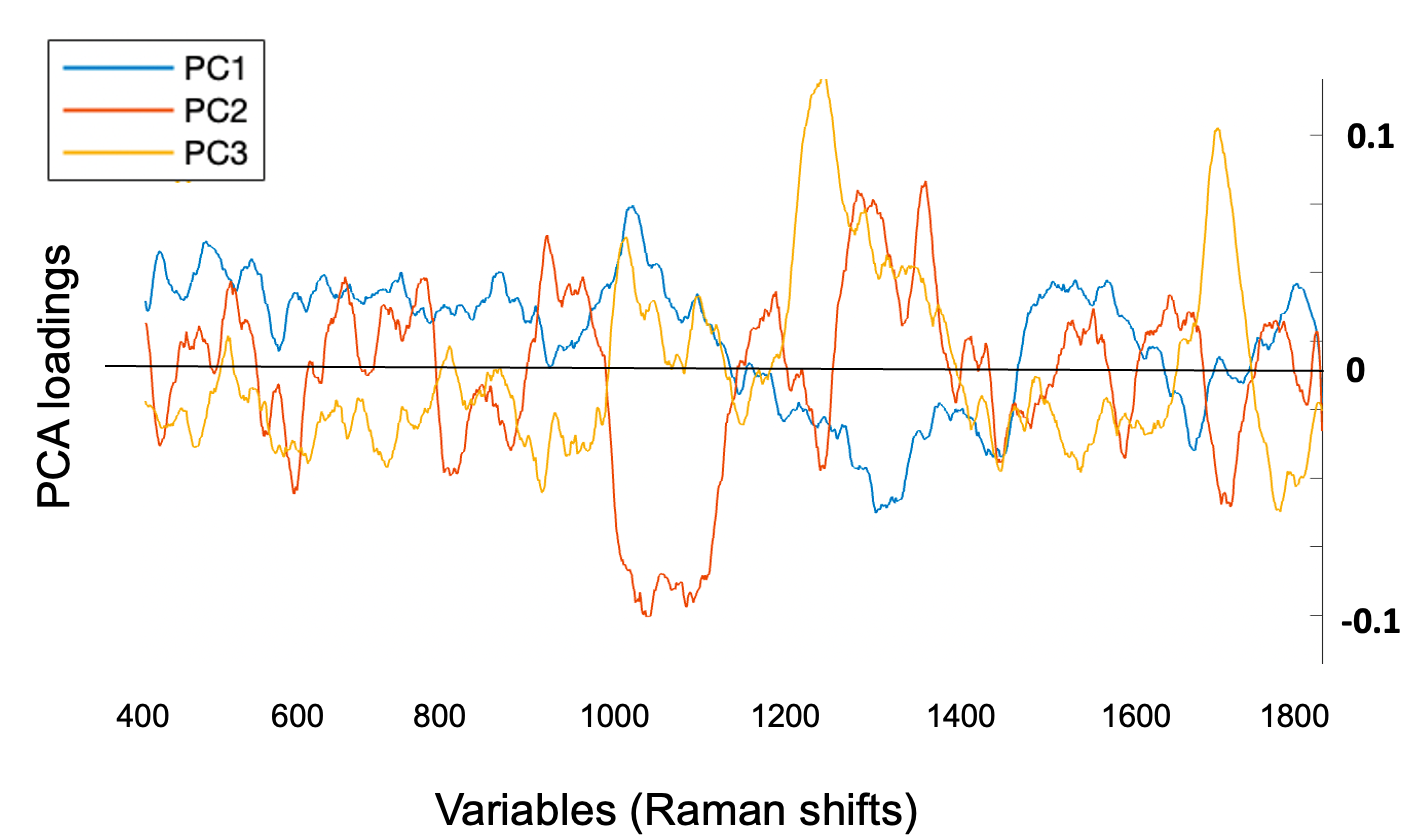


**Figure S4. PCA loadings plot of glioma tissue discrimination.** The PCA loadings of the three largest PCs are plotted against the variables (Raman shifts). This representation permits the identification of each PC’s contribution to the variance observed between the grades at each point of the spectrum. The importance of the 950-1200 cm^-1^ and 1250-1350 cm^-1^ regions are further confirmed.


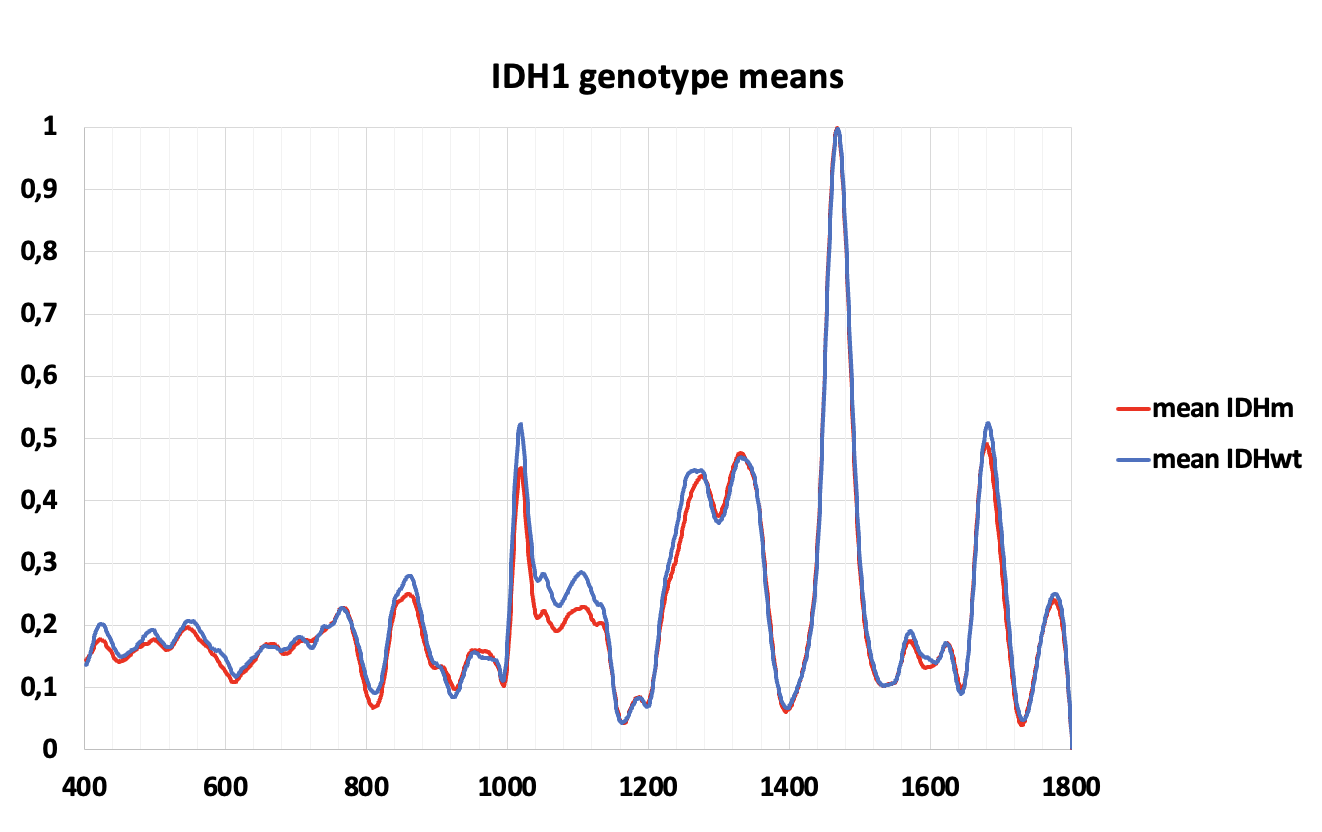


**Figure S5. IDH-mutated and IDH-wild type mean Raman spectra.**

**
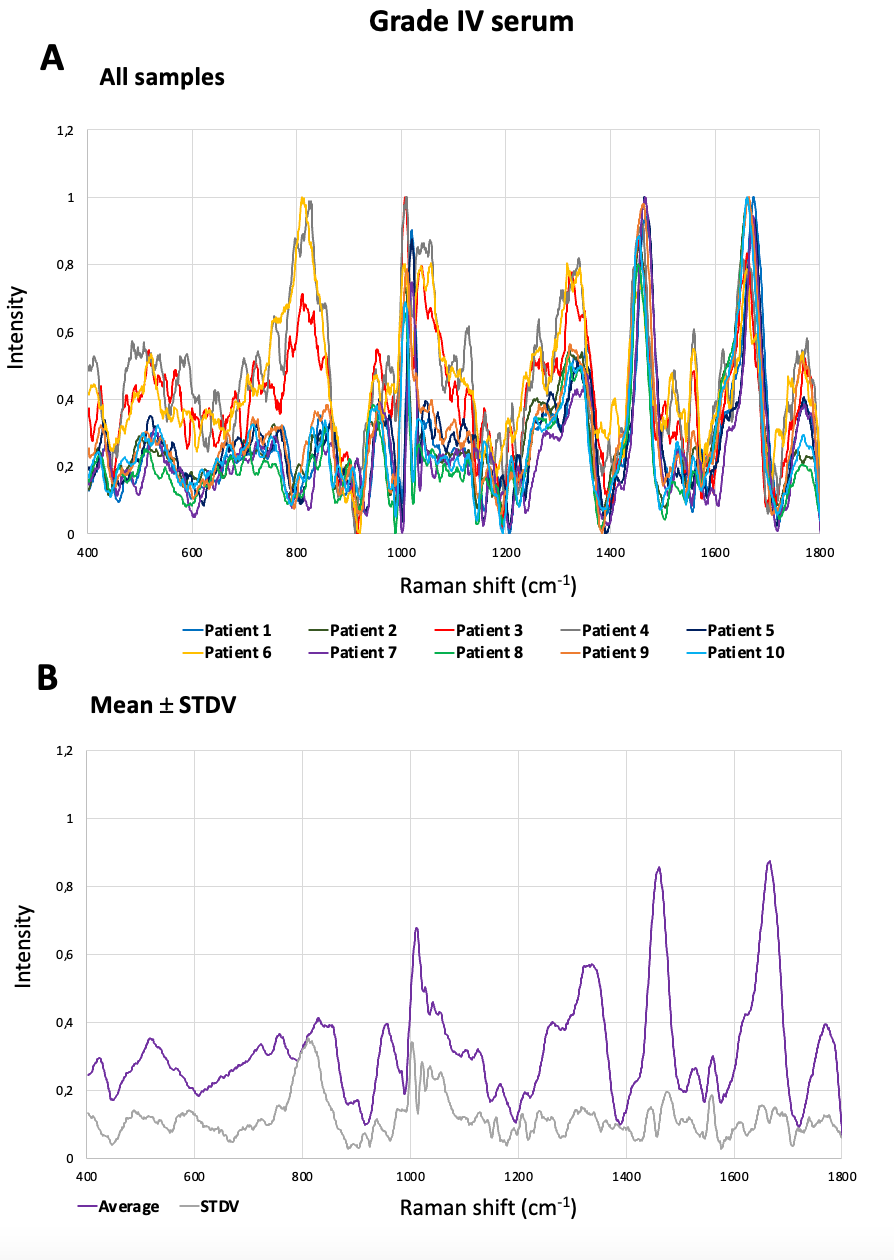
**

**Figure S6. Mean serum spectra of grade IV patients.** **(A)** All mean spectra for each patient (each sample). **(B)** Mean spectra (in purple) for grade IV with the standard deviation (in grey).


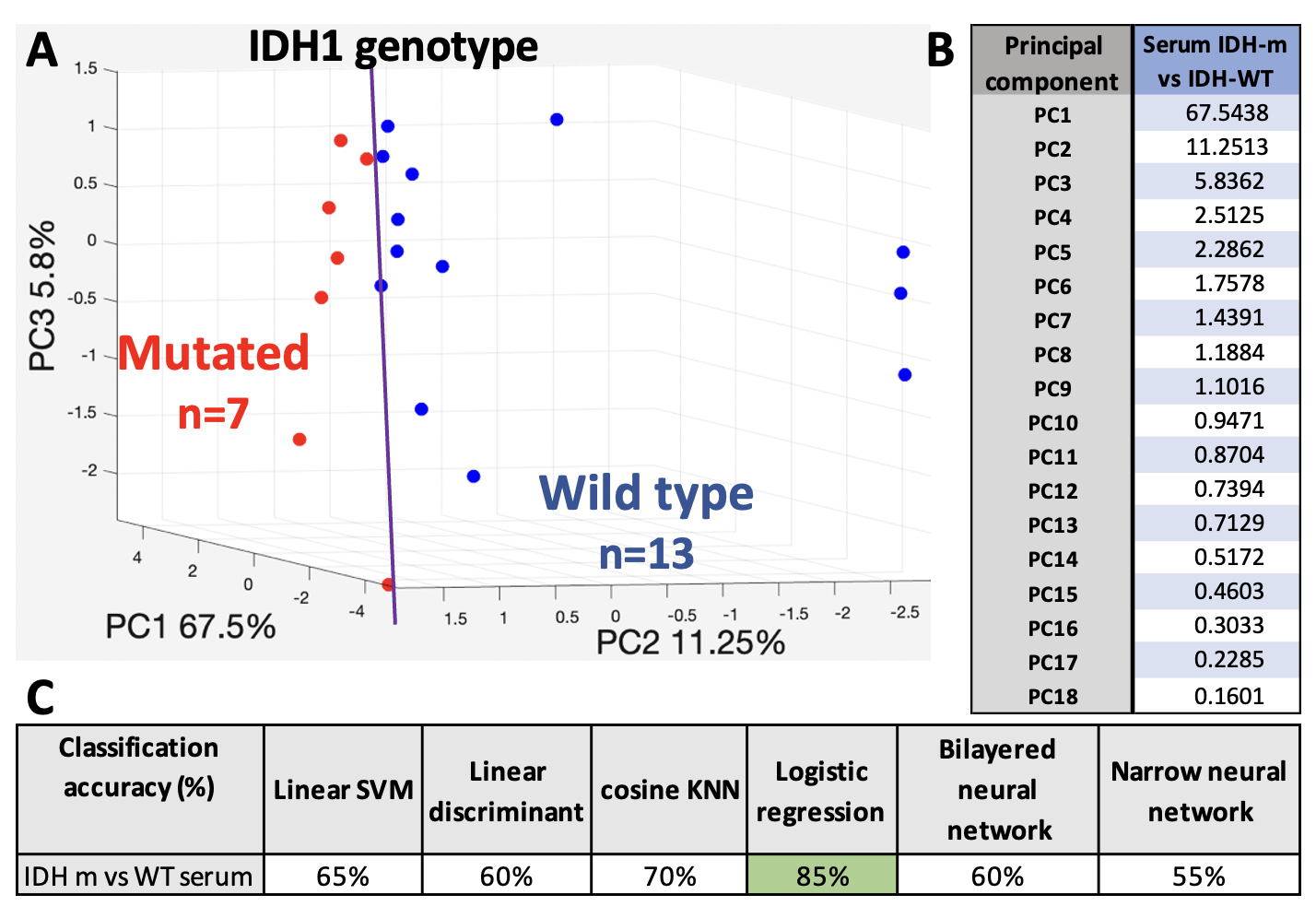


**Figure S7. Discrimination between IDH1-mutated and IDH1-wild type patients in serum. (A)** 3D PCA plot of IDH1-mutated (n=7) and IDH1-wild type (n=13) patients (grade III and IV). The purple line indicates the discrimination trend between the two group and was determined subjectively by eye. **(B)** Percentages of variance explained by each PC. **(C)** Classification accuracy for different models of classification learning. Accuracy rate above 75% are highlighted in green.

**Table S1. Patients’ characteristics.**

**Table S2. Percentages of variance explained between tissue samples.** The percentages of variance explained between the samples by each PC for each comparison are given for the tissue and the serum PCAs. CTRL: controls.

| **Peak (cm^-1^)** | **Component(s) assigned** | **Peak (cm^-1^)** | **Component(s) assigned** |
| --- | --- | --- | --- |
| **446-476** | cholesterol, proteins | **1095** | mannose |
| **498** | nucleic acid | **1120-1121** | GAG, glucose |
| **500-550** | proteins | **1127** | proteins |
| **589** | amide I band | **1129** | lipids |
| **636-638** | tyrosine, lactose | **1131-1139** | mannose |
| **640-646** | proteins | **1157** | carotenoids |
| **670** | haemoglobin | **1206-1207** | proteins |
| **676** | DNA | **1225** | haemoglobin |
| **700-703** | proteins, cholesterol | **1242** | GAG |
| **725-729** | proteins, nucleic acid | **1230-1306** | amide III, proteins, collagen, lipids |
| **743-790** | DNA, proteins, haemoglobin | **1300** | proteins |
| **818** | collagen | **1263** | lipids |
| **823** | tyrosine | **1313** | phospholipids |
| **843** | glucose | **1322** | collagen, proteins |
| **857** | proteins, collagen, glycans, GAG | **1331-1338** | nucleic acid, proteins, glucose |
| **870** | glucose | **1358** | cytochrome C |
| **880-890** | tryptophan, galactosamine | **1365** | tryptophan |
| **910-911** | glucose | **1378** | DNA |
| **933** | proteins | **1380** | glucose |
| **935-940** | proteins, collagen | **1402** | phospholipids |
| **959** | proteins | **1441-1445** | collagen, phospholipids |
| **997** | glucose | **1450** | proteins |
| **1003-1005** | phenylalanine, collagen | **1521** | carotenoids |
| **1000-1200** | glucose | **1541-1546** | amide II, cytochrome C |
| **1032** | proteins | **1573-1585** | nucleic acid, proteins, haemoglobin |
| **1064-1068** | lipids | **1602-1607** | proteins |
| **1074** | phospholipids, collagen | **1654-1657** | amide I, collagen, lipids |
|  |  | **1732** | lipids |

**Table S3. General database from the literature.** Main peaks with their corresponding assignments from the literature.
